# A novel method using an ultra-thin cholangioscope and through the cholangioscope snare to remove an embedded internally migrated pancreatic stent from a previously normal pancreatic duct

**DOI:** 10.1055/a-2615-2304

**Published:** 2025-07-29

**Authors:** Daniyal Baig, Javaid Iqbal, Venkata Lekharaju, Mohamed Korani, Sajjad Mahmood

**Affiliations:** 1 5293 Department of Gastroenterology/Endoscopy, Manchester University NHS Foundation Trust, Manchester, United Kingdom

The use of pancreatic stents is integral for hepatopancreatobiliary and pancreaticobiliary endoscopy, primarily for post-ERCP pancreatitis prophylaxis and management of pancreatic strictures 1 . While their application is crucial for patient outcomes, stent migration [occurring in up to 5.2% of cases], poses significant challenges 2 3 . Failure to retrieve proximally migrated stents endoscopically can lead to serious complications, with 10–17% of cases requiring surgical intervention 3 . Advanced endoscopic solutions are crucial to mitigate these risks.

We report a novel case of successful retrieval of a proximally migrated pancreatic stent using an ultra-thin Leinzett cholangioscope (2.6 mm) and SpySnare – the first documented use of this method for PD stent removal in the UK.

A 29-year-old man presented with cholangitis secondary to common bile duct stones. During ERCP, a pancreatic stent was placed following an inadvertent wire passage into the pancreatic duct. The entire stent subsequently migrated proximally deep into pancreas, requiring advanced endoscopic retrieval. Initial pancreatoscopy confirmed the migration ( Video 1 , Fig. 1 ). A 0.025-mm visiglide guidewire facilitated entry into the pancreatic duct with the SpyGlass cholangioscope (Boston Scientific). However, the stent was embedded around the genu causing a short inflammatory stricture preventing further advancement. An 8 cm (5 mm diameter) pancreatic stent was then deployed to dilate the duct.

Endoscopic retrieval of a proximally migrated, embedded pancreatic stent using an ultra-thin Leinzett cholangioscope and SpySnare. The video demonstrates the stepwise approach, including initial cholangioscopy, guidewire-assisted ductal access, dilation with a Hurricane balloon, and successful stent extraction using SpySnare.Video 1

**Fig. 1:**
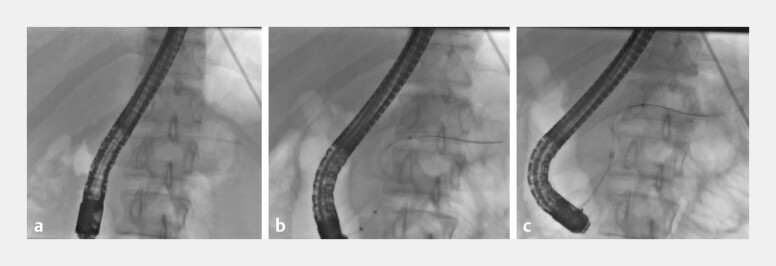
Fluoroscopic images during the initial endoscopic assessment. **a** Initial embedded pancreatic stent. **b** Guidewire placed into the pancreatic duct. **c** SpyGlass cholangioscope advancing towards the pancreatic duct unable to pass due to stricture.

On the second attempt, the Hurricane balloon dilator enabled improved access; yet, the retrieval remained unsuccessful because of the embedded position of the stent. A Boston 5 Fr × 7 cm stent was then used to remodel the duct ( Fig. 2 ). During a third attempt, an ultra-thin Leinzett Lan-EP-3516 cholangioscope and SpySnare were used ( Fig. 3 **a** ). The SpySnare engaged the proximal portion of the stent allowing precise manipulation for successful extraction ( Fig. 3 **b** ).

**Fig. 2:**
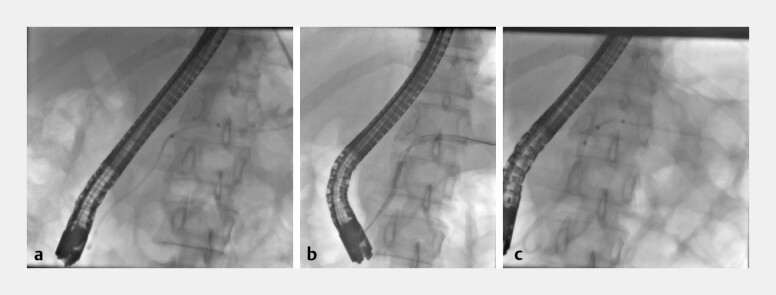
Fluoroscopic images demonstrating the second retrieval attempt. **a** Two pancreatic stents (migrated stent and stent placed on the previous scope to help remodel stricture). **b** Balloon dilation with a Hurricane dilator. **c** Remodeling of the PD with a Boston 5 Fr × 7 cm stent.

**Fig. 3:**
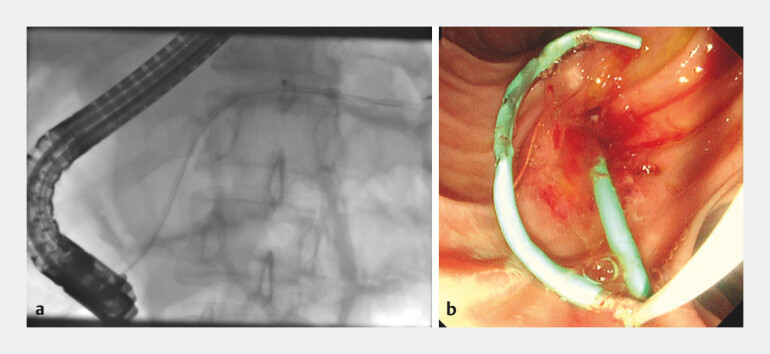
**a** Fluoroscopic image showing SpySnare engagement of the proximally migrated pancreatic stent. **b** Endoscopic view of the successfully retrieved stent.

This case reports the utility of ultra-thin cholangioscopy and SpySnare in overcoming challenges associated with internally migrated embedded pancreatic stents. This case presents the potential of these methods to reduce the need for surgical interventions and improve outcomes in tertiary centres.

Endoscopy_UCTN_Code_CPL_1AK_2AD

## Notes

Endoscopy E-Videos https://eref.thieme.de/e-videos
*E-Videos* is an open access online section of the journal *Endoscopy* , reporting on interesting cases and new techniques in gastroenterological endoscopy. All papers include a high-quality video and are published with a Creative Commons CC-BY license. Endoscopy E-Videos qualify for HINARI discounts and waivers and eligibility is automatically checked during the submission process. We grant 100% waivers to articles whose corresponding authors are based in Group A countries and 50% waivers to those who are based in Group B countries as classified by Research4Life (see: https://www.research4life.org/access/eligibility/ ). This section has its own submission website at https://mc.manuscriptcentral.com/e-videos .
